# Arbitrary-order intrinsic virtual element method for elliptic equations on surfaces

**DOI:** 10.1007/s10092-021-00418-5

**Published:** 2021-06-21

**Authors:** Elena Bachini, Gianmarco Manzini, Mario Putti

**Affiliations:** 1 Department of Geosciences and Department of Mathematics “Tullio Levi-Civita”, University of Padua, Padua , Italy; 2 Present Address: Department of Mathematics, Institute of Scientific Computing, TU Dresden, Dresden, Germany; 3 Istituto di Matematica Applicata e Tecnologie Informatiche del Consiglio Nazionale delle Ricerche, Pavia, Italy; 4 Department of Mathematics “Tullio Levi-Civita”, University of Padua, Padua, Italy

**Keywords:** Surface PDEs, Geometrically intrinsic operators, Virtual element method, Polygonal mesh, high-order methods, 65N30, 58J05

## Abstract

We develop a geometrically intrinsic formulation of the arbitrary-order Virtual Element Method (VEM) on polygonal cells for the numerical solution of elliptic surface partial differential equations (PDEs). The PDE is first written in covariant form using an appropriate local reference system. The knowledge of the local parametrization allows us to consider the two-dimensional VEM scheme, without any explicit approximation of the surface geometry. The theoretical properties of the classical VEM are extended to our framework by taking into consideration the highly anisotropic character of the final discretization. These properties are extensively tested on triangular and polygonal meshes using a manufactured solution. The limitations of the scheme are verified as functions of the regularity of the surface and its approximation.

## Introduction

Surface partial differential equations of elliptic and parabolic types are often used for the simulation of diverse phenomena in many fields of applications, for example in biology, atmospheric dynamics, and image processing [53, 55]. One of the main motivation that prompted this work is related to the modeling of gravity-driven flows in earth-sciences, such as, e.g., flood forecasting, landslide and debris flow dynamics, avalanche simulations [6, 20, 39, 41]. The numerical solution of surface PDEs has seen wide-spread interest in the last few years with different approaches being proposed, including continuous Finite Element Methods (FEM), discontinuous Galerkin (DG), finite volumes, trace-FEMs, etc. [4, 6, 7, 33, 36, 41, 54, 55]. A recent survey of surface FEM was published in [37], where both steady and moving surfaces are considered, the latter finding a unifying theory in [38].

Although FE-based approaches are very successful in the numerical treatment of surface PDEs, they share the limitation that an explicit form of the basis functions is required in the formulation of the method, and thus are restricted mostly to triangular/quadrilateral elements. This restriction is overcome by the Virtual Element Method (VEM) that was designed from the very beginning to work on generally shaped elements with high order of accuracy. In fact, in the VEM approach (*i*) it is possible to decompose the computational domain into very general polygonal elements; (*ii*) an explicit form of the basis functions is not required; (*iii*) approximation of arbitrary order and arbitrary regularity are straightforward in two and three dimensions. VEM was originally developed as a variational reformulation of the *nodal* mimetic finite difference (MFD) method [10, 13, 24, 47] for solving diffusion problems on unstructured polygonal meshes. A survey on the MFD method can be found in the review paper [45] and the research monograph [12]. The scheme inherits the flexibility of the MFD method with respect to the admissible meshes and this feature is well reflected in the many significant applications that have been developed so far, see, for example, [3, 5, 8, 9, 14–19, 27, 29, 30, 32, 51, 56, 57, 64].Because of its origins, VEM is intimately connected with other FE-based approaches. The connection between the VEM and finite elements on polygonal/polyhedral meshes is thoroughly investigated in [26, 35, 46], between VEM and discontinuous skeletal gradient discretizations in [35], and between the VEM and the BEM-based FEM method in [28]. VEM was originally formulated in [11] as a conforming FEM for the Poisson problem, and was later extended to convection-reaction-diffusion problems with variable coefficients in [15]. However, the VEM technology has seen so far very few applications to surface PDEs, and only with first-order polynomial accuracy [42].

One of the major difficulties in the high-order numerical solution of surface PDEs is the achievement of a consistent approximation of both the geometry and the PDE. The work of [34] develops a general technique for high-order polygonal approximation of a smooth manifold, but this method requires the explicit knowledge of the distance function within the tubular neighborhood of the surface. The task of approximating this distance function to high order is still an open problem [50]. A recent approach based on this idea was presented in [4], where the authors study a high-order (up to four) DG scheme based on a piecewise polynomial approximation of the surface triangulation. However, extensions to polygonal grids with high polynomial orders have not yet been addressed. While VEM provides an ideal framework to work at high-order on generally shaped cells, according to [42] the main difficulty is the high-order approximation of the surface, limiting their current developments to polynomials of order one. The same authors suggest the use of the approach in [34] to extend their VEM scheme to higher order polynomials, without however eliminating the difficulty of the approximation to a consistent order of the distance function.

In this paper, we develop a novel VEM-based approach for the solution of elliptic surface PDEs that works at all polynomial orders. We avoid the difficulties related to high-order surface approximation by employing intrinsic geometry and following the approach described in [7] to adapt the virtual element technology to the surface PDE. Using this approach, we first rewrite the partial differential equation in covariant form in such a way that the geometric information, essentially the metric tensor, is completely encoded in the equation itself. As a consequence, the numerical scheme can be constructed directly on the two-dimensional local chart where the surface parametrization is defined, thus enabling the full exploitation of the VEM machinery. Here, we restrict our attention to the case when the surface is defined by a single chart, a case of great interest for example in gravity-driven flows on terrain surfaces, such as water flow and sediment transport in mountain areas [20, 21, 39, 40, 52]. In principle, our proposed approach can be applied to the more general situation of a surface defined by an atlas if the transition between charts is done with care by enforcing proper smoothness as described in [44]. This is shown by testing our approach on the sphere by using the well-known charts arising from the stereographical projection.

Our method starts from a partition of the surface into polygons with curvilinear edges, assuming that the parametrization of the surface is known at relevant quadrature points. Proceeding from the covariant PDE, we construct a high-order scheme exploiting the ability of the VEM approach to discretize problems that are anisotropic and with spatially variable coefficients [15]. In practice, we re-define the PDE on a local coordinate system using intrinsic geometric quantities and operators, which contain explicitly the metric information deriving from the surface. Then, all the VEM projection operators are calculated using this local coordinate system and the knowledge of the parametrization is used to define the needed quantities, thus incurring in no explicit geometric error. Hence the final scheme is defined on a planar two-dimensional domain (the surface chart) and all the available machinery to achieve high-order on polygonal cells can be exploited. The price we pay is that now the PDE contains the anisotropic metric tensor and all the coefficients vary in space as a function of the regularity of the surface. The virtual element method has proved its efficiency in handling these situations [49] and can be implemented directly in this two-dimensional setting. In addition, with our approach the convergence theory extends straight-forwardly to surface problems without additional efforts.

Our development of the intrinsic VEM proceeds as follows. In Sect. 2, we describe the local reference system of choice and the geometric setting. Next, we define the differential operators and the corresponding PDE in covariant form. In Sect. 3, we summarize the VEM adopted in this work and discuss the necessary adaptations to the problem at hand. The final Sect. 4 reports the results of extensive numerical experiments that assess the effectiveness, accuracy, and robustness of the proposed approach.

## The surface partial differential equation and its Galerkin discretization

**Notation** Throughout the paper, we use the standard definition and notation of Sobolev spaces, norms and seminorms (see [1]), which can be directly extended to a compact manifold $\varGamma$ (see [63]). Given $\omega$ an open and bounded subset of $\mathbb {R} ^d$, $d=2,3$, we denote with $L^{p} (\omega )$ and $W^{k,p} (\omega )$ the Lebesgue and Sobolev spaces, with $W^{k,2} (\omega )= H^{k} (\omega )$ the classical Hilbert space. Norms and seminorms in $H^{k}(\omega )$ are denoted by $||\cdot ||_{H^{k}(\omega )}$ and $|\cdot |_{H^{k}(\omega )}$, respectively, and $(\cdot ,\cdot )_{\omega }$ denotes the inner product in $L^2(\omega )$. We omit the subscript in the inner product notation when $\omega$ is the whole computational domain. In a few situations, for the sake of clarity, we may prefer to use the integral notation of the inner product.

Consider a compact surface $\varGamma \subset {\mathbb {R}}^{3}$ with boundary $\partial \varGamma$ over which the following elliptic partial differential equation is defined:

1 $$\begin{aligned} -\Delta _{\scriptscriptstyle \mathcal {G} }u+ \left\langle {\mathbf {w}},{{\nabla }_{\scriptscriptstyle \mathcal {G} }u} \right\rangle _{\scriptscriptstyle \mathcal {G} } +\gamma \; u&= f\qquad \hbox {on~} \varGamma ,\nonumber \\ u&= 0\qquad \hbox {on~} \partial \varGamma , \end{aligned}$$

where the solution $u: \varGamma \rightarrow \mathbb {R}$ is a scalar function defined on the surface, $\mathbf {w}: \varGamma \rightarrow {\mathbb {R}}^{2}$ is a given divergence-free velocity field tangent to the surface, the function $\gamma : \varGamma \rightarrow \mathbb {R}$ is a non-negative reaction coefficient. We denote by $\Delta _{\scriptscriptstyle \mathcal {G} }$ and ${\nabla }_{\scriptscriptstyle \mathcal {G} }$ the Laplace-Beltrami and the tangential gradient operators, respectively, and by $\left\langle {\cdot },{\cdot } \right\rangle _{\scriptscriptstyle \mathcal {G} }$ the intrinsic scalar product. These operators will be given precise definitions depending on the chosen coordinate system. Classically, we assume $f\in H^{-1}( \varGamma )$, $\mathbf {w}\in [ W^{1,\infty } ( \varGamma )]^2$, and $\gamma -\tfrac{1}{2}{\nabla }_{\scriptscriptstyle \mathcal {G} }\cdot \mathbf {w}>0$. Here we consider homogeneous Dirichlet problems with the more general boundary conditions described in [25].

The variational formulation of Eq. (1) reads:

### Problem 1

(Intrinsic variational formulation)

Find $u\in H^{1}_{0} ( \varGamma )$ such that

2 $$\begin{aligned} a(u, v ) + b(u, v ) + c(u, v ) = F( v ) \quad \forall \, v \in H^{1}_{0} ( \varGamma ), \end{aligned}$$

where the bilinear forms $a(\cdot ,\cdot ) ,\, b(\cdot ,\cdot ) ,\, c(\cdot ,\cdot ) :\, H^{1} ( \varGamma )\times H^{1} ( \varGamma )\rightarrow \mathbb {R}$ are given by

$$\begin{aligned} a(u, v ) := \; \int _{ \varGamma } \left\langle {{\nabla }_{\scriptscriptstyle \mathcal {G} }u},{{\nabla }_{\scriptscriptstyle \mathcal {G} } v } \right\rangle _{\scriptscriptstyle \mathcal {G} } , \qquad b(u, v ) := \int _{ \varGamma } \left\langle {\mathbf {w}},{{\nabla }_{\scriptscriptstyle \mathcal {G} }u} \right\rangle _{\scriptscriptstyle \mathcal {G} } v , \qquad c(u, v ) := \int _{ \varGamma }\gamma \,u\, v , \end{aligned}$$

and the right-hand side linear functional $F(\cdot ):\,H^1_0( \varGamma )\rightarrow \mathbb {R}$ is given by

$$\begin{aligned} F( v ) := \int _{ \varGamma }f\, v . \end{aligned}$$

### Remark 1

The well-posedness of Problem 1 follows from the application of the Lax-Milgram theorem since the classical theory of elliptic equations can be extended to surface PDEs in a straight-forward manner. In particular, due to the coercivity of the bilinear form $a(\cdot ,\cdot )$, the continuity of the bilinear forms $a(\cdot ,\cdot )$, $b(\cdot ,\cdot )$, and $c(\cdot ,\cdot )$ and the linear functional $F(\cdot )$, and under the assumption that $\gamma -\tfrac{1}{2}{\nabla }_{\scriptscriptstyle \mathcal {G} }\cdot \mathbf {w}>0$, the solution $u$ exists and is unique and belongs to $H^1_0(\varGamma )$ if $f\in H^{-1}(\varGamma )$. For a detailed description of the properties, well-posedness, and regularity of the variational problem on manifolds we refer to [43] and to [62, 63].

The discrete approximation of this problem reads as follows:

### Problem 2

(Intrinsic discrete Galerkin approximation) Find $u_{h}\in V^{h}_{k}$ such that

3 $$\begin{aligned} a_{h}(u_{h},v_{h}) + b_{h}(u_{h},v_{h}) + c_{h}(u_{h},v_{h}) = F_{h}(v_{h}) \qquad \forall v_{h}\in V^{h}_{k}, \end{aligned}$$

where $V^{h}_{k}$ is the functional space that provides a conforming approximation of $H^{1}_{0} ( \varGamma )$ in the virtual element setting, and $u_{h}$, $a_{h}(\cdot ,\cdot )$, $b_{h}(\cdot ,\cdot )$, $c_{h}(\cdot ,\cdot )$, and $F_{h}(\cdot )$ are the virtual element approximations to $u$, $a(\cdot ,\cdot )$, $b(\cdot ,\cdot )$, $c(\cdot ,\cdot )$ and $F(\cdot )$.

These mathematical objects are defined and discussed in the next sections.

### Geometrical setting

We assume that the surface $\varGamma$ is $C^{m}$ regular, i.e.:

#### Definition 1

(Regular Surface) A connected set $\varGamma \subset {\mathbb {R}}^{3}$ is a $C^{m}$ *regular or embedded* surface if for all $\mathbf {p} \in \varGamma$ there exists an open subset ${U} \subseteq {\mathbb {R}}^{2}$ and a map ${\phi _{\mathbf{p}}}: {U} \rightarrow {\mathbb {R}}^{3}$ of class $C^{m}$, $m\in \mathbb N \cup \{\infty \}$, such that:

i ${\phi _{\mathbf{p}}}( {U} )\subseteq \varGamma$ is an open neighborhood of $\mathbf {p} \in \varGamma$;
ii ${\phi _{\mathbf{p}}}$ is a homeomorphism with its image (i.e., there exists an open neighborhood of $\mathbf {p}$, ${V} \subseteq {\mathbb {R}}^{3}$ such that ${\phi _{\mathbf{p}}}( {U} )= {V} \cap \varGamma$);
iii The differential ${\mathrm{d}}{\phi _{\mathbf{p}}}: {\mathbb {R}}^{2} \rightarrow {\mathbb {R}}^{3}$ is injective in ${U}$ (i.e., it has maximum rank, in our case 2).

The map ${\phi _{\mathbf{p}}}$ is the *local parametrization* of $\varGamma$ centered in $\mathbf {p}$ and we denote with ${\phi _{\mathbf{p}}}^{-1}: {V} \cap \varGamma \rightarrow {U}$ its inverse map, called the *local chart*, in $\mathbf {p}$. The set ${\phi _{\mathbf{p}}}( {U} )\subset \varGamma$ is called a *coordinate neighborhood*, while $( s^{\scriptscriptstyle {1}} _{\scriptscriptstyle {\mathbf {q}}} , s^{\scriptscriptstyle {2}} _{\scriptscriptstyle {\mathbf {q}}} )$ are the *local coordinates* of any point $\mathbf {q}\in {\phi _{\mathbf{p}}}( {U} )$.

#### Remark 2

Throughout the paper we assume that $\varGamma$ is contained in only one chart. This is not a limitation under the assumption of $C^{\infty }$ regularity of $\varGamma$ (or $C^{m}$ with *m* sufficiently large) since we can always find compatible local parametrizations covering $\varGamma$. Indeed, given two points $\mathbf {p}$ and $\mathbf {q}\in \varGamma$ with local parametrizations ${\phi _{\mathbf{p}}}$ and $\phi _{\mathbf {q}}$ such that ${U} _{ \mathbf {p} }\cap {U} _{\mathbf {q}}\ne \emptyset$, the transition map ${\phi _{\mathbf{p}}}\circ \phi _{\mathbf {q}} ^{-1}$ is a $C^{\infty }$ (or $C^{m}$) diffeomorphism. Thus, it is always possible to find an atlas for $\varGamma$ formed by appropriate charts that maintains all the required continuity properties. A proper selection of these charts is fundamental to obtain a numerically well-conditioned reference system in our approach. For an example of a constructive methodology for the definition of smooth multi-charts see [44].

For simplicity, from now on we will drop the subscripts $\mathbf {p}$ and $\mathbf {q}$ in both the local coordinates $\mathbf {s} =( s^{\scriptscriptstyle {1}} , s^{\scriptscriptstyle {2}} )$ and the global Cartesian coordinates $\mathbf {x} =( x^{\scriptscriptstyle {1}} , x^{\scriptscriptstyle {2}} , x^{\scriptscriptstyle {3}} )$. In summary, we have the following explicit definitions of these transformations:

$$\begin{aligned} \phi : {U}&\rightarrow {V} \cap \varGamma& \quad \phi ^{-1}: {V} \cap \varGamma&\rightarrow {U} \\ \mathbf {s}&\mapsto \mathbf {x}&\mathbf {x}&\mapsto \mathbf {s} \end{aligned}$$

We want to choose a coordinate system to give a workable meaning to the partial differential equation and related differential operators. We define the local reference system following the approach in [6, 7]. To this aim, we compute the pair of tangent vectors $\{ \hat{\mathbf {t}}_{1} ( \mathbf {p} ), \hat{\mathbf {t}}_{2} ( \mathbf {p} )\}$ on the tangent plane $T_{\scriptscriptstyle { \mathbf {p} }} \varGamma$:

$$\begin{aligned} \hat{\mathbf {t}}_{i} ( \mathbf {p} ) = \left( \frac{ \partial x^{\scriptscriptstyle {1}} }{ \partial s^{\scriptscriptstyle {i}} }, \frac{ \partial x^{\scriptscriptstyle {2}} }{ \partial s^{\scriptscriptstyle {i}} }, \frac{ \partial x^{\scriptscriptstyle {3}} }{ \partial s^{\scriptscriptstyle {i}} } \right) , \qquad i = 1,2 . \end{aligned}$$

This pair is orthogonalized via Gram-Schmidt, yielding the orthogonal frame $\{ \mathbf {t}_{1} , \mathbf {t}_{2} \}$. The ensuing metric tensor is given by:

4 $$\begin{aligned} \mathcal {G} := \left( \begin{array}{ccc} \left\| \mathbf {t}_{1} ( \mathbf {p} ) \right\| ^2 &{} 0 \\ 0 &{} \left\| \mathbf {t}_{2} ( \mathbf {p} ) \right\| ^2\\ \end{array} \right) . \end{aligned}$$

The associated scalar product between two vectors $\mathbf {u}$ and $\mathbf {v}$ is given by $\left\langle { \mathbf {u} },{ \mathbf {v} } \right\rangle _{\scriptscriptstyle \mathcal {G} } ={ \mathbf {u} }\cdot { \mathcal {G} \, \mathbf {v} }={ \mathcal {G} \, \mathbf {u} }\cdot { \mathbf {v} }$, where “$\cdot$” is the canonical $\mathbb {R} ^2$ scalar product. Tensor $\mathcal {G}$ represents the realization of the first fundamental form with respect to the chosen reference system (chart). For a $C^{m}$-regular surface (see definition 1), the determinant ${\text {det}} \mathcal {G}$ of the metric tensor is a well-defined and bounded function, and the metric tensor itself is coercive, i.e., it is symmetric and positive-definite and has a symmetric and positive-definite inverse. In other words, we can find constants $g_*$ and $g^*$ such that [31]:

5 $$\begin{aligned} g_* \left\| \mathbf {u} \right\| ^2\le \left\langle { \mathbf {u} },{ \mathcal {G} \, \mathbf {u} }\right\rangle \le g^* \left\| \mathbf {u} \right\| ^2, \end{aligned}$$

where $$ \left\| \mathbf {u} \right\| ^2= \mathbf {u} \cdot \mathbf {u} $$.

We can now write the intrinsic differential operators with respect to the local coordinate system, and we collect the appropriate definitions in the following proposition, which we state without proof:

#### Proposition 1

(Intrinsic Differential Operators) Given $f : \varGamma \rightarrow \mathbb {R}$ a scalar differentiable function on $\varGamma$ and denoting with ${\nabla }$ and ${\nabla }\cdot$ the gradient and divergence operators in $\mathbb {R} ^2$, the *intrinsic* differential operators expressed in the local coordinate system are given by the following expressions:

- The *intrinsic* gradient of $f$ is:
  6 $$\begin{aligned} {\nabla }_{\scriptscriptstyle \mathcal {G} } f = \mathcal {G} ^{-1}{\nabla } f \, . \end{aligned}$$
- The *intrinsic* Laplace-Beltrami operator of $f$ is:
  7 $$\begin{aligned} \Delta _{\scriptscriptstyle \mathcal {G} } f = {\nabla }_{\scriptscriptstyle \mathcal {G} }\cdot {\nabla }_{\scriptscriptstyle \mathcal {G} } f = \frac{1}{\sqrt{{\text {det}} \mathcal {G} }} {\nabla }\cdot \left( \sqrt{{\text {det}} \mathcal {G} }\, \mathcal {G} ^{-1} {\nabla } f \right) . \end{aligned}$$

We would like to recall that our reference frame is covariant and thus scalar products must act on vectors written in contravariant components. This applies both to velocity vector and to the divergence operator as well.

#### Remark 3

We can use the orthonormal reference frame $\mathbf {e}_{1} , \mathbf {e}_{2}$ as a base for $\mathbb {R} ^2$ with which we can express differential operators and vector quantities. In this case we need to take into consideration the Jacobian matrix $\mathbf {J}=[ \mathbf {t}_{1} , \mathbf {t}_{2} ]$ of the parametrization, and recall that $\mathcal {G} = \mathbf {J}^{{\tiny T}}\mathbf {J}$. This applies in particular to the velocity vector field $\mathbf {w}$, which can be written as:

$$\begin{aligned} \mathbf {w}= [w^1,w^2]^{{\tiny T}} = \mathcal {G} ^{-1/2}[w_{(1)},w_{(2)}]^{{\tiny T}}= \mathcal {G} ^{-1/2}\,\hat{\mathbf {w}}\,, \end{aligned}$$

where $\hat{\mathbf {w}}=[w_{(1)}, w_{(2)}]^T$ is the velocity vector written with respect to $\mathbf {e}_{1} , \mathbf {e}_{2}$.

In this setting, we can give the definition of the integral of a function over a surface as follows:

#### Definition 2

Let $f : \varGamma \rightarrow \mathbb {R}$ be a continuous function defined on a regular surface $\varGamma$, which we assume contained in the image of a local parametrization $\phi : {U} \rightarrow \varGamma$.

The $f$ *on* $\varGamma$ is given by

$$\begin{aligned} \int _{ \varGamma } f = \int _{ \phi ^{-1}( \varGamma )}( f \circ \phi )\,\sqrt{{\text {det}} \mathcal {G} } \;{\, \text {d}}{ \mathbf {s} } . \end{aligned}$$

We can relate any function $f : \varGamma \rightarrow \mathbb {R}$ to a specific coordinate system using the above coordinate transformations, i.e.:

$$\begin{aligned} f ( \mathbf {x} )= f \circ \phi ( \mathbf {s} ) = \hat{f} ( \mathbf {s} )\;. \end{aligned}$$

In the following we will make use only of the local coordinate system and will write $f ( \mathbf {s} )$ omitting the hat symbol.

The classical tools deriving from Stokes theorems hold with the intrinsic operators without any modification. In particular, the intrinsic Green formula can be stated as in the following lemma.

#### Lemma 1

(Intrinsic Green formula) Let $\varGamma \subset {\mathbb {R}}^{3}$ be a surface with smooth boundary $\partial \varGamma$ and given two functions $u\in C^{2} ( \varGamma )$ and $v \in H^{1} ( \varGamma )$, then:

8 $$\begin{aligned} \int _{ \varGamma } \left\langle {{\nabla }_{\scriptscriptstyle \mathcal {G} }u},{{\nabla }_{\scriptscriptstyle \mathcal {G} } v } \right\rangle _{\scriptscriptstyle \mathcal {G} } = -\int _{ \varGamma }\Delta _{\scriptscriptstyle \mathcal {G} }u\; v + \int _{ \partial \varGamma } \left\langle {{\nabla }_{\scriptscriptstyle \mathcal {G} }u},{\mathbf {\mu }} \right\rangle _{\scriptscriptstyle \mathcal {G} } v \;, \end{aligned}$$

where $\mathbf {\mu }: \varGamma \rightarrow {\mathbb {R}}^{2}$ denotes the vector tangent to $\varGamma$ and normal to $\partial \varGamma$ with components written with respect to the local reference frame (i.e. $\mathbf {\mu }=\mu ^1 \mathbf {t}_{1} +\mu ^2 \mathbf {t}_{2}$).

In view of remark 3, we reformulate the bilinear forms $a(\cdot ,\cdot )$, $b(\cdot ,\cdot )$, and $c(\cdot ,\cdot )$ the linear functional $F(\cdot )$ of the intrinsic variational formulation (2) on the chart $\phi ^{-1}( \varGamma )$ through:

$$\begin{aligned} a(u, v ) = \int _{ \phi ^{-1}( \varGamma )}\left( \sqrt{{\text {det}} \mathcal {G} }\, \mathcal {G} ^{-1}\right) {\nabla }u\cdot {\nabla } v {\, \text {d}} { \mathbf {s} } \,,\qquad b(u, v ) = \int _{ \phi ^{-1}( \varGamma )}\left( \sqrt{{\text {det}} \mathcal {G} }\, \mathcal {G} ^{-1/2}\right) \hat{\mathbf {w}}\cdot {\nabla }u\; v {\, \text {d}}{ \mathbf {s} } \,, \end{aligned}$$

and

$$\begin{aligned} c(u, v ) = \int _{ \phi ^{-1}( \varGamma )}\sqrt{{\text {det}} \mathcal {G} }\,\gamma \,u\, v {\, \text {d}} { \mathbf {s} } \,,\qquad \quad F( v )= \int _{ \phi ^{-1}( \varGamma )}\sqrt{{\text {det}} \mathcal {G} }f\, v {\, \text {d}} { \mathbf {s} } \,. \end{aligned}$$

Therefore, the intrinsic variational formulation (2) is equivalent to solving the advection-diffusion-reaction equation in variational form:

9 $$\begin{aligned} \int _{ \phi ^{-1}( \varGamma )} \Big (\mathsf {K}{\nabla }u\cdot {\nabla } v +\widetilde{\mathbf {w}}\cdot {\nabla }u+\widetilde{\gamma }u v \Big ) {\, \text {d}} { \mathbf {s} } = \int _{ \phi ^{-1}( \varGamma )}\widetilde{ f } v {\, \text {d}} { \mathbf {s} } , \end{aligned}$$

where the equation coefficients are defined by

$$\begin{aligned} \mathsf {K}= \sqrt{{\text {det}} \mathcal {G} }\, \mathcal {G} ^{-1}\,, \qquad \widetilde{\mathbf {w}} = \left( \sqrt{{\text {det}} \mathcal {G} }\, \mathcal {G} ^{-1/2}\right) \hat{\mathbf {w}}\,,\qquad \widetilde{\gamma } = \sqrt{{\text {det}} \mathcal {G} }\,\gamma \,,\qquad \widetilde{ f } = \sqrt{{\text {det}} \mathcal {G} }\, f . \end{aligned}$$

The problem as above formulated is still well-posed and maintains all the properties listed in remark 1. Indeed, since our surface is assumed to be regular, there exist two positive constants $c_{_{ \mathcal {G} }}$ and $C_{_{ \mathcal {G} }}$ such that ${c_{_{ \mathcal {G} }}\le \sqrt{{\text {det}} \mathcal {G} }\le C_{_{ \mathcal {G} }}}$ and

10 $$\begin{aligned} \kappa _*{|u|^{2}}_{{H^{1}}({\varGamma })}\le a(u,u) \le \kappa ^*{|u|^{2}}_{{H^{1}}({\varGamma })}, \end{aligned}$$

where $\kappa _*=c_{_{ \mathcal {G} }}g^*$ and $\kappa ^*=C_{_{ \mathcal {G} }}g_*$, and $g_*$ and $g^*$ are the constants introduced in (5). The coercivity of $a(\cdot ,\cdot )$ with respect to the $H^1$-norm follows immediately by noting that ${|u|}_{{H^{1}}({\varGamma })}\le \left\| u \right\| _{{H^{1}}({\varGamma })}$ and applying the Poincaré inequality in $H^{1}_{0} ( \varGamma )$. Moreover, there exist two positive constants $w_{\max }$ and $\gamma _{\max }$ such that $\left\| \widetilde{\mathbf {w}} \right\| _{\infty } \le C_{_{ \mathcal {G} }} \left\| \mathbf {w} \right\| _{\infty } \le w_{\max }$ and $\widetilde{\gamma }\le {{C}_{{ \mathcal {G} }}}\,\gamma \le \gamma _{\max }$.

## The virtual element method

**Fig. 1:**
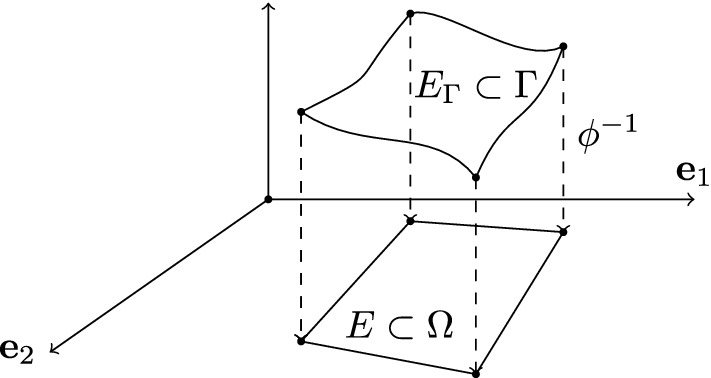
Example of surface polygonal element $E_{ \varGamma }$ in $\varGamma$ and corresponding (planar) element $E$ in $\Omega$

In this section, we discuss the virtual element approximation of problem 2. The numerical method that we use in this work is based on refs. [2, 11, 15], which define optimal approximations of the finite dimensional spaces on polygonal meshes when the equation coefficients are variable in space. As already observed in remark 2, we work on a single coordinate neighborhood $\Omega = \phi ^{-1}( \varGamma )$ and consider the (global) parametrization $\phi :\Omega \rightarrow \varGamma$. We start from a partition of $\varGamma$ formed by surface polygonal elements $E_{ \varGamma }$ with edges denoted by $e_{ \varGamma }$. Through the parametrization $\phi$, we can associate the partition of $\varGamma$ with a partition of $\Omega$ formed by elements $E$ and possibly curvilinear edges $e$. Because of the regularity assumption on the surface, every element $E_{ \varGamma }$ is in a one-to-one relation with one and only one polygonal element $E$ in $\Omega$. To avoid curvilinear edges, we use the surface vertices of $E_{ \varGamma }$ to define the vertices of the polygons $E$ in $\Omega$ through the inverse parametrization and connect them with straight segments to define the partition of $\Omega$. This procedure maintains the above one-to-one relationship between elements $E_{ \varGamma }$ in $\varGamma$ and $E$ in $\Omega$ (see Fig. 1).

In addition, any function in $E_{ \varGamma }$ can be expressed in $E$ by composition with the inverse parametrization. Thus, all the local functional spaces of interest can be defined indifferently on $E_{ \varGamma }$ or $E$. The definitions of the building blocks of the virtual element method is done in $\Omega$ using standard two-dimensional Cartesian coordinates. These constructions are needed to evaluate the surface bilinear forms and the right-hand side linear functional of the weak formulation (2) by a careful use of the metric tensor.

**The conforming virtual element space** Let $\mathcal {T}=\{\Omega _{h}\}_{h}$ be a set of decompositions $\Omega _{h}$ of the computational domain $\Omega$ into a finite set of nonoverlapping polygonal elements $E$. The subindex label $h$ is the maximum of the diameters of the mesh elements, i.e., $h_{E}=\sup _{ \mathbf {s} ', \mathbf {s} ''\in E}| \mathbf {s} '- \mathbf {s} ''|$. Each element $E$ has a nonintersecting boundary denoted by $\partial E$ formed by straight edges $e$, center of gravity $\mathbf {s} _{E}$ and area $\left| E \right|$. A few regularity assumptions are needed on the mesh family $\{\Omega _{h}\}$ to prove the convergence of the VEM and derive the error estimates in the $L^2$ and $H^1$ norms. We present these assumptions at the end of this section where we briefly discuss the convergence of the proposed VEM.

Let $k\ge 1$ be an integer number and $E\in \Omega _{h}$ a generic mesh element. The *conforming virtual element space *$V^{h}_{k}$ *of order* $k\ge 1$ *built on mesh* $\Omega _{h}$ is obtained by gluing together the local approximation spaces denoted by $V^{h}_{k}(E)$:

11 $$\begin{aligned} V^{h}_{k}:=\Big \{\,v_{h}\in H^1_0(\Omega )\,:\,{v_{h}}_{|{E}}\in V^{h}_{k}(E) \,\,\,\forall E\in \Omega _{h}\,\Big \}. \end{aligned}$$

The local virtual element space $V^{h}_{k}(E)$ is defined in accordance with the *enhancement strategy* introduced in [2]:

12 $$\begin{aligned} V^{h}_{k}(E) = \bigg \{\, v_{h}\in H^1(E)\cap C^{0}(\overline{E})\,:\, {v_{h}}_{|{\partial E}}\in C^{0}(\partial E),\, {v_{h}}_{|{e}}\in \mathbb {P}_{k}(e)\,\forall e\subset \partial E,\, \Delta v_{h}\in \mathbb {P}_{k}(E),\,\nonumber \\ \int _{E}(v_{h}-\Pi ^{\nabla ,E}_{k}v_{h})\,m\,d \mathbf {s} =0 \,\,\forall m\in \mathbb {P}_{k}(E)\backslash \mathbb {P}_{k-2}(E) \,\bigg \}, \end{aligned}$$

where $\mathbb {P}_{k}(E)$ and $\mathbb {P}_{k}(e)$ denote the polynomial spaces of degree at most *k* defined over an element $E$ or an edge $e$, respectively. By definition, each space $V^{h}_{k}(E)$ contains $\mathbb {P}_{k}(E)$ and the global space $V^{h}_{k}$ is a conforming subspace of $H^1_0(\Omega )$. The definition of the virtual element bilinear forms $a_{h}(\cdot ,\cdot )$, $b_{h}(\cdot ,\cdot )$, and $c_{h}(\cdot ,\cdot )$, and the forcing term $F_{h}(\cdot )$ requires the definition of the elliptic and orthogonal projections operators.

**Elliptic projection** The *elliptic projection operator* $\Pi ^{\nabla ,E}_{k}:H^1(E)\rightarrow \mathbb {P}_{k}(E)$ can be defined for any $v_{h}\in V^{h}_{k}(E)$ as:

13 $$\begin{aligned} \int _{E}\nabla \Pi ^{\nabla ,E}_{k}v_{h}\cdot \nabla q{\, \text {d}}{ \mathbf {s} }&= \int _{E}\nabla v_{h}\cdot \nabla q{\, \text {d}}{ \mathbf {s} } \quad \forall q\in \mathbb {P}_{k}(E), \end{aligned}$$

14 $$\begin{aligned} \int _{\partial E}\big (\Pi ^{\nabla ,E}_{k}v_{h}-v_{h}\big ){\, \text {d}}{ \sigma }= 0. \end{aligned}$$

Equation (14) allows the removal of the kernel of the gradient operator. The elliptic projection operator $\Pi ^{\nabla ,E}_{k}$ is a polynomial-preserving operator, i.e., $\Pi ^{\nabla ,E}_{k}q=q$ for every $q\in \mathbb {P}_{k}(E)$. One of its major property is that the projection $\Pi ^{\nabla ,E}_{k}v_{h}$ of any virtual element function $v_{h}\in V^{h}_{k}(E)$ is computable from the degrees of freedom of $v_{h}$ [11], which are defined as follows.

The degrees of freedom of the virtual element function $v_{h}\in V^{h}_{k}(E)$ are given by the set of values:

**(D1)**: For $k\ge 1$, the values of $v_{h}$ at the vertices of $E$;
**(D2)**: For $k\ge 2$, the values of $v_{h}$ at the $k-1$ internal nodes of the *k*-th Gauss-Lobatto formula on every $e\in \partial E$;
**(D3)**: For $k\ge 2$, the cell moments of $v_{h}$ of order up to $k-2$ on element $E$:
  15 $$\begin{aligned} \frac{1}{ \left| E \right| }\int _{E}v_{h}\,m\,d \mathbf {s} , \,\,\forall m\in \mathcal {M}_{k-2}(E), \end{aligned}$$

where $\mathcal {M}_{k-2}(E)$ is the set of scaled monomials that span the linear space of polynomials of degree up to $k-2$. These set of values are unisolvent in $V^{h}_{k}(E)$, cf. [11], and thus, every virtual element function is uniquely identified by them. The degrees of freedom of a virtual element function in the global space $V^{h}_{k}$ are given by collecting the elemental degrees of freedom **(D1)**–**(D3)**. Their unisolvence in $V^{h}_{k}$ is an immediate consequence of their unisolvence in every elemental space $V^{h}_{k}(E)$. 

**Orthogonal projections** From the degrees of freedom of a virtual element function $v_{h}\in V^{h}_{k}(E)$ we can also compute the orthogonal projections $\Pi ^{0,E}_{k}v_{h}$ and $\Pi ^{0,E}_{k-1}\nabla v_{h}$, cf. [2]. In fact, the definition of the orthogonal projection $\Pi ^{0,E}_{k}v_{h}$ reads as

16 $$\begin{aligned} \int _{E}\Pi ^{0,E}_{k}v_{h}\,q{\, \text {d}}{ \mathbf {s} } = \int _{E}v_{h}\,q{\, \text {d}}{ \mathbf {s} } \qquad \forall q\in \mathbb {P}_{k}(E). \end{aligned}$$

The right-hand side is the integral of $v_{h}$ against the polynomial $q$, and is computable from the degrees of freedom **(D3)** of $v_{h}$ when $q$ is a polynomial of degree up to $k-2$, and from the moments of $\Pi ^{\nabla ,E}_{k}v_{h}$ when $q$ is a polynomial of degree $k-1$ and *k*, cf. (12). Clearly, the orthogonal projection $\Pi ^{0,E}_{k-1}v_{h}$ is also computable.

In turn, using the definition of the orthogonal projection $\Pi ^{0,E}_{k-1}\nabla v_{h}$ and integrating by parts, we find that

17 $$\begin{aligned} \int _{E}\Pi ^{0,E}_{k-1}\nabla v_{h}\cdot \mathbf {q}{\, \text {d}}{ \mathbf {s} } = \int _{E}\nabla v_{h}\cdot \mathbf {q}{\, \text {d}}{ \mathbf {s} } = -\int _{E}v_{h}\nabla \cdot \mathbf {q}{\, \text {d}}{ \mathbf {s} } + \sum _{e\in \partial E}\int _{e}v_{h}\mathbf {n}_{E,e}\cdot \mathbf {q}{\, \text {d}}{ \sigma } \end{aligned}$$

for every $\mathbf {q}\in \left[ \mathbb {P}_{k-1}(E)\right] ^{2}$, where $\mathbf {n}_{E,e}$ denotes the unit outward vector orthogonal to the boundary edge $e\in \partial E$. The first integral on the (last) right-hand side is computable from the degrees of freedom **(D3)**, i.e., from the moments of $v_{h}$ against the polynomials of degree $k-2$ over $E$. The edge integrals are computable from the degrees of freedom **(D1)**–**(D2)** because we can compute the trace of $v_{h}$ on each edge by interpolating these nodal values. 

**The virtual element bilinear forms** Following the VEM gospel, we write the discrete bilinear forms $a_{h}(\cdot ,\cdot )$, $b_{h}(\cdot ,\cdot )$ and $c_{h}(\cdot ,\cdot )$ as the sum of elemental contributions

18 $$\begin{aligned} a_{h}(u_{h},v_{h}) = \sum _{E\in \Omega _{h}}a^{E}_{h}(u_{h},v_{h}),\quad b_{h}(u_{h},v_{h}) = \sum _{E\in \Omega _{h}}b^{E}_{h}(u_{h},v_{h}),\quad c_{h}(u_{h},v_{h}) = \sum _{E\in \Omega _{h}}c^{E}_{h}(u_{h},v_{h}). \end{aligned}$$

The bilinear forms $a^{E}_{h}(u_{h},v_{h})$, $b^{E}_{h}(u_{h},v_{h})$ and $c^{E}_{h}(u_{h},v_{h})$ on each element $E$ are given by

19 $$\begin{aligned} a^{E}_{h}(u_{h},v_{h})&= \int _{E}\sqrt{\det { \mathcal {G} }} \mathcal {G} ^{-1}\,\Pi ^{0,E}_{k-1}\nabla u_{h}\cdot \Pi ^{0,E}_{k-1}\nabla v_{h}{\, \text {d}}{ \mathbf {s} } + S^{E}_{h}\Big ( \big (I-\Pi ^{\nabla ,E}_{k}\big )u_{h}, \big (I-\Pi ^{\nabla ,E}_{k}\big )v_{h}\Big ), \end{aligned}$$

20 $$\begin{aligned} b^{E}_{h}(u_{h},v_{h})&= \int _{E}\sqrt{\det { \mathcal {G} }} \mathcal {G} ^{-1/2}\hat{\mathbf {w}}\cdot \Pi ^{0,E}_{k-1}{\nabla }u_{h}\;\Pi ^{0,E}_{k-1}v_{h} {\, \text {d}}{ \mathbf {s} } , \end{aligned}$$

21 $$\begin{aligned} c^{E}_{h}(u_{h},v_{h})&= \int _{E}\sqrt{\det { \mathcal {G} }}\gamma \Pi ^{0,E}_{k-1}u_{h}\,\Pi ^{0,E}_{k-1}v_{h}{\, \text {d}}{ \mathbf {s} } . \end{aligned}$$

The bilinear form $S^{E}_{h}(\cdot ,\cdot )$ in the definition of $a^{E}_{h}(\cdot ,\cdot )$ provides the stability term and can be any symmetric positive definite bilinear form defined on $E$ for which there exist two positive constants $c_*$ and $c^*$ such that

22 $$\begin{aligned} c_*a^{E}(v_{h},v_{h}) \le S^{E}_{h}(v_{h},v_{h}) \le c^*a^{E}(v_{h},v_{h}) \quad \forall v_{h}\in V^{h}_{k}(E)\text {~with~}\Pi ^{\nabla ,E}_{k}v_{h}=0. \end{aligned}$$

Note that $S^{E}_{h}(\cdot ,\cdot )$ must scale like the restriction of $a(\cdot ,\cdot )$ on the mesh element $E$. Also, the stabilization term in the definition of $a^{E}_{h}(\cdot ,\cdot )$ gives a zero contribution if one of its two entries is a polynomial of degree (at most) *k* since $\Pi ^{\nabla ,E}_{k}$ is a projection on the polynomial space. In this work, we consider two possible implementations of the stability term:

- The choice originally provided in [11], which is sometimes called the “*dofi-dofi stabilization*” in the virtual element literature, and reads as
  23 $$\begin{aligned} S^{E}_{h}(v_{h},w_{h}) = \sum _{i=1}^{N^{\tiny \text {DOF}}}\text {DOF}_i(v_{h})\text {DOF}_i(w_{h}), \end{aligned}$$
  where $\text {DOF}_i(\cdot )$ is the map between a virtual function and its degrees of freedom;
- The formula proposed in [48], which is sometimes called the “*D-recipe stabilization*” in the virtual element literature, and reads as
  24 $$\begin{aligned} S^{E}_{h}(v_{h},w_{h}) = \sum _{i=1}^{N^{\tiny \text {DOF}}}\mathcal {A}_{ii}\text {DOF}_i(v_{h})\text {DOF}_i(w_{h}), \end{aligned}$$
  where $\mathcal {A}$ is the matrix resulting from the implementation of the first term in the bilinear form $a^{E}_{h}(\cdot ,\cdot )$:
  25 $$\begin{aligned} \mathcal {A}_{ij}:=\int _{E}\sqrt{\det { \mathcal {G} }} \mathcal {G} ^{-1}\,\Pi ^{0,E}_{k-1}\nabla \varphi _i\cdot \Pi ^{0,E}_{k-1}\nabla \varphi _j{\, \text {d}}{ \mathbf {s} } , \end{aligned}$$
  where $\varphi _i$ (and $\varphi _j$) are the “canonical” basis functions generating $V^{h}_{k}(E)$, i.e., the functions whose $i-th$ (or $j-th$) degree of freedom is equal to 1 and all other degrees of freedom are 0. We note that these basis function are unknown in the virtual element framework, but their projections $\Pi ^{0,E}_{k-1}\nabla \varphi _i$ (and $\Pi ^{0,E}_{k-1}\nabla \varphi _j$) are computable.

The stabilization term, and, in particular, condition (22), is designed in order that $a^{E}_{h}(\cdot ,\cdot )$ satisfies the two fundamental properties:

- *k*-*consistency*: for all $v_{h}\in V^{h}_{k}$ and for all $q\in \mathbb {P}_{k}(E)$ it holds
  26 $$\begin{aligned} a^{E}_{h}(v_{h},q) = a^{E}(v_{h},q); \end{aligned}$$
- *Stability*: there exist two positive constants $\alpha _*,\,\alpha ^*$, independent of $h$ and $E$, such that
  27 $$\begin{aligned} \alpha _*a^{E}(v_{h},v_{h}) \le a^{E}_{h}(v_{h},v_{h}) \le \alpha ^*a^{E}(v_{h},v_{h})\quad \forall v_{h}\in V^{h}_{k}. \end{aligned}$$

**The virtual element forcing term** To approximate the right-hand side of (3), we split the term into the sum of elemental contributions and approximate every local linear functional by means of the orthogonal projection $\Pi ^{0,E}_{k}v_{h}$:

28 $$\begin{aligned}&F_{ h }(v_{h}) = \sum _{E\in \Omega _{h}}\big (f,\Pi ^{0,E}_{k}v_{h}\big )_{E} \text {~~where~~}\big (f,\Pi ^{0,E}_{k}v_{h}\big )_{E} = \int _{E}\sqrt{\det { \mathcal {G} }}\,f\,\Pi ^{0,E}_{k}v_{h}{\, \text {d}}{ \mathbf {s} } . \end{aligned}$$

With these definitions the VEM scheme in problem 2 is completely determined. 

**Convergence properties** The numerical analysis of the scheme requires the following hypotheses on the mesh, typical of VEM methods.

### Assumption 1

(Mesh regularity assumptions) There exists a positive constant $\varrho$ independent of $h$ (and, hence, of $\Omega _{h}$) such that

(i) Every element $E$ of every mesh $\Omega _{h}$ is star-shaped with respect to a disk with radius $\ge \varrho h_{E}$;
(ii) Every edge $e\in \partial E$ has length $h_{e}\ge \varrho h_{E}$.

The star-shapedness property (*i*) implies that the polygonal elements are *simply connected* subsets of $\mathbb {R} ^{2}$. In turn, the scaling assumption (*ii*) implies that the number of edges in each elemental boundary is uniformly bounded over the whole mesh family $\{\Omega _{h}\}$.

The following theorem summarizes the results for the virtual element approximation in problem 2. The proof of these results found in [15] is easily extended to our setting. Indeed, we choose to write the theorem in terms of the chart $\Omega$ and its discretization $\Omega _{h}$, but it can be written equivalently in terms of the surface $\varGamma$ and its discretization $\varGamma _{h}$, since the norms of the parametrization and its inverse are uniformly bounded by hypothesis.

### Theorem 1

Let $u\in H^{k+1}(\Omega )\cap H^1_0(\Omega )$, be the solution to the variational problem 2 on a convex domain $\Omega$ with ${f\in H^{k}(\Omega )}$. Let $u_{h}\in V^{h}_{k}$ be the solution of the virtual element method (3) on every mesh of a mesh family $\{\Omega _{h}\}$ satisfying the mesh regularity assumption 1. Then, a strictly positive constant $C$ independent of $h$ exists such that

- The $H^1$-error estimate holds:
  29 $$\begin{aligned} || u-u_{h}||_{H^1(\Omega )}\le Ch^{k}\left( |u|_{H^{k+1}(\Omega )} + |f|_{H^{k}(\Omega )} \right) ; \end{aligned}$$
- The $L^2$-error estimate holds:
  30 $$\begin{aligned} || u-u_{h}||_{L^2(\Omega )}\le Ch^{k+1}\left( |u|_{H^{k+1}(\Omega )} + |f|_{H^{k}(\Omega )} \right) . \end{aligned}$$

The constant $C$ may depend on the coefficient bounds $\kappa _{*}$, $\kappa ^*$, $w_{\max }$ and $\gamma _{\max }$, the stability constants $\alpha _*$ and $\alpha ^*$, the mesh regularity constant $\varrho$, the size of the computational domain $\left| \Omega \right|$, and the approximation degree *k*.

The approximate solution $u_{h}$ is not explicitly known inside the elements. Consequently, in the numerical experiments of Sect. 4, we approximate the error norms as follows:

$$\begin{aligned} || u-u_{h}||_{H^1(\Omega )}\approx || u-\Pi ^{0}_{k}u_{h}||_{H^1(\Omega _{h})} \quad \text {and}\quad || u-u_{h}||_{L^2(\Omega )}\approx || u-\Pi ^{0}_{k}u_{h}||_{L^2(\Omega )}. \end{aligned}$$

Here, $\Pi ^{0}_{k}u_{h}$ is the global projector on the space of discontinuous polynomials of degree at most *k* built on mesh $\Omega _{h}$, and $|| u-\Pi ^{0}_{k}u_{h}||_{H^1(\Omega _{h})}$ is the norm in the broken Sobolev space $H^1(\Omega _{h})$ that is defined by summing the $H^1(E)$-norms of each element $E$. Operator $\Pi ^{0}_{k}u_{h}$ is obtained by taking the elemental $L^2$-orthogonal projections $\Pi ^{0,E}_{k}u_{h}$ in every mesh element $E$, which are computable from the degrees of freedom of $u_{h}$, so that ${\big (\Pi ^{0}_{k}u_{h}\big )}_{|{E}}=\Pi ^{0,E}_{k}\big ({u_{h}}_{|{E}}\big )$.

## Numerical results

**Fig. 2:**
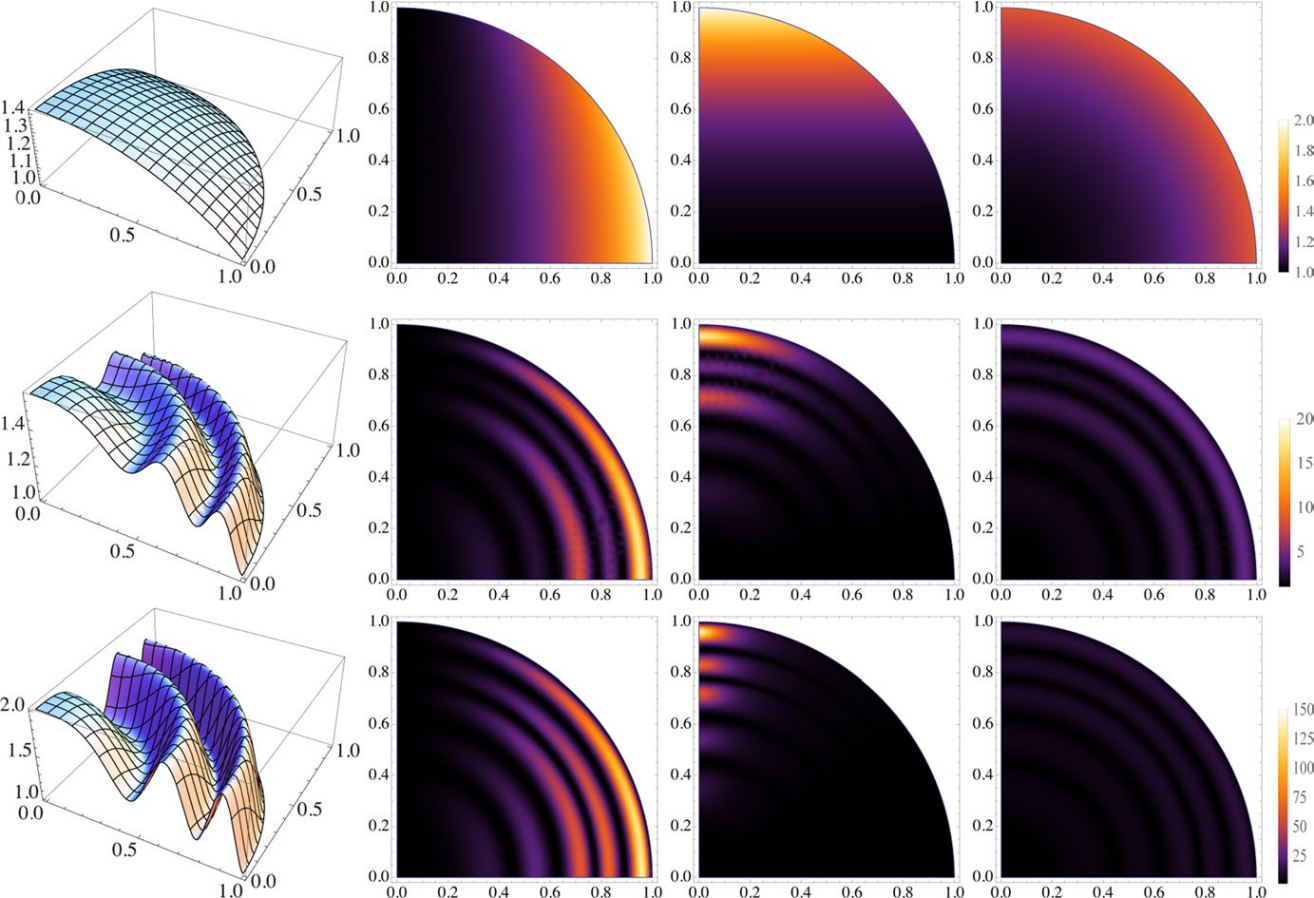
Surfaces and metric components used in the numerical experiments (see Eq. (31)). The columns show the spatial behavior of the surface $\varGamma$, $g_{\scriptscriptstyle {11}}$, $g_{\scriptscriptstyle {22}}$, and $\sqrt{{\text {det}} \mathcal {G} }$ ($r=2$, $a=0$ first row; $r=2$, $a=0.5$, $k=5$ second row; $r=2$, $a=2.0$, $k=5$ third row). Note the completely different color scales between the case $a=0$ and the cases $a>0$, where the metric tensor displays strong anisotropy ($g_{\scriptscriptstyle {11}}\approx 20 \,(150)$ where $g_{\scriptscriptstyle {22}}\approx 1$). The choice of an orthogonal reference frame ensures that the principal directions of anisotropy (the eigenvectors of $\mathcal {G}$) in the second order diffusion term do not vary in space

In this section we present numerical results on synthetic test cases to support the statements of the previous sections by means of experimental evidence. Our test cases are grouped into four main categories. The first two sets of experiments, Test Cases 1 and 2, are aimed at showing the correctness of our implementation and the order of convergence of the proposed VEM scheme up to fourth order of accuracy. In the third set of experiments, Test Case 3, we explore the limits of the VEM approach as the metric tensor $\mathcal {G}$ becomes more and more anisotropic (large condition numbers) as a function of the regularity of the surface. Finally, Test Case 4 considers the use of stereographic projection to build a two chart atlas for the sphere to show the applicability of our approach in a multi-chart case.

In the first three experiments we consider the surface provided by the graph of the following height function, a simple trigonometric perturbation of a portion of a sphere embedded in $\mathbb {R} ^3$:

31 $$\begin{aligned} x^{\scriptscriptstyle {3}} = \mathcal {H} ( x^{\scriptscriptstyle {1}} , x^{\scriptscriptstyle {2}} )=\sqrt{r - ( x^{\scriptscriptstyle {1}} )^2 - ( x^{\scriptscriptstyle {2}} )^2 + a \cos ^2\left( k\frac{\pi }{2} (( x^{\scriptscriptstyle {1}} )^2 + ( x^{\scriptscriptstyle {2}} )^2)\right) } \; , \end{aligned}$$

where *r* is the radius of the sphere, and *a* and *k* are the amplitude and the frequency of the cosine trigonometric perturbation. We use the Monge parametrization given by $\phi =\{ x^{\scriptscriptstyle {1}} = s^{\scriptscriptstyle {1}} , x^{\scriptscriptstyle {2}} = s^{\scriptscriptstyle {2}} , x^{\scriptscriptstyle {3}} = \mathcal {H} ( x^{\scriptscriptstyle {1}} , x^{\scriptscriptstyle {2}} )\}$ and work on the single chart represented by the domain $\Omega =\{( s^{\scriptscriptstyle {1}} , s^{\scriptscriptstyle {2}} ): s^{\scriptscriptstyle {1}} , s^{\scriptscriptstyle {2}} \ge 0 \hbox { and } \sqrt{( s^{\scriptscriptstyle {1}} )^2+( s^{\scriptscriptstyle {2}} )^2}\le 1\}$. For $r\rightarrow 1$ the metric tensor $\mathcal {G}$ tends to become singular as one of the two tangent vectors increases indefinitely at the boundary of the surface, leading to large spectral condition numbers $\kappa ( \mathcal {G} )$. Analogously, the condition number of $\mathcal {G}$ increases when the frequency *k* and the amplitude *a* are increased. Fig. 2 shows the three-dimensional plot of the surface in the left panel, and the spatial distributions of $g_{\scriptscriptstyle {11}}$, $g_{\scriptscriptstyle {22}}$, and $\sqrt{{\text {det}} \mathcal {G} }$, respectively, in the next three columns. The rows are relative to the case $r=2$, with the sphere ($a=0$) shown in the top, while the trigonometric deformation of the sphere is shown in the middle row for the case $a=0.5$ and $k=5$, and in the bottom row for the case $a=2$ and $k=5$. In this latter cases we note that $\mathcal {G}$ has a sinusoidal behavior with $g_{\scriptscriptstyle {11}}\approx 1$ in the regions where $g_{\scriptscriptstyle {22}}\approx 20(150)$, leading to $\kappa ( \mathcal {G} )\approx 20(150)$. Note that $\mathcal {G}$ does not enter the reaction term and has a small effect in the advection term, as it amounts to a rotation and a stretching of the advective field. On the other hand, it has a large effect on the coercivity of the diffusion bilinear form, and thus we concentrate on the latter. The condition number of the VEM stiffness matrix $\mathcal {A}$ can be bounded by [22, 58]:

$$\begin{aligned} \kappa (\mathcal {A})\le \max _{ \mathbf {s} \in \Omega }\left( \sqrt{{\text {det}} \mathcal {G} ( \mathbf {s} )}\, \kappa ( \mathcal {G} ^{-1}( \mathbf {s} )) \right) \kappa (\mathcal {A}_{\scriptscriptstyle {\text {LAP}}}), \end{aligned}$$

where $\kappa (\mathcal {A}_{\scriptscriptstyle {\text {LAP}}})$ is the condition number of the VEM stiffness matrix of Laplace equation. In our case, we have that:

$$\begin{aligned} \sqrt{{\text {det}}( \mathcal {G} )}\;\kappa ( \mathcal {G} ^{-1}) =\sqrt{\frac{\max \{g_{\scriptscriptstyle {11}},g_{\scriptscriptstyle {22}}\}^{3}}{\min \{g_{\scriptscriptstyle {11}},g_{\scriptscriptstyle {22}}\}}}. \end{aligned}$$

Note that, $\sqrt{{\text {det}}( \mathcal {G} )}\;\kappa ( \mathcal {G} ^{-1})\ge 1$ is smooth although possibly unbounded when $r\rightarrow 1$ (or *k* and *a* are large), as mentioned above. We would like to remark that this is not a contraddiction of Eq. (10), but rather a consequence of the fact that we use a single Monge parametrization. The presence of the metric tensor in the equation always deteriorates, possibly drastically, the condition number of the system matrix.

In Test Case 4, we consider Laplace equation (i.e., $\mathbf {w}=0$ and $\gamma =0$) on $\varGamma =S^2$ and use two parametrizations, one for the northern and one for the southern hemispheres, given by:

$$\begin{aligned} \phi _{N} ( s^{\scriptscriptstyle {1}} , s^{\scriptscriptstyle {2}} ) =\left( \frac{2 s^{\scriptscriptstyle {1}} }{1+( s^{\scriptscriptstyle {1}} )^2+( s^{\scriptscriptstyle {2}} )^2}, \frac{2 s^{\scriptscriptstyle {2}} }{1+( s^{\scriptscriptstyle {1}} )^2+( s^{\scriptscriptstyle {2}} )^2}, \frac{1-( s^{\scriptscriptstyle {1}} )^2-( s^{\scriptscriptstyle {2}} )^2}{1+( s^{\scriptscriptstyle {1}} )^2+( s^{\scriptscriptstyle {2}} )^2}\right) =( x^{\scriptscriptstyle {1}} , x^{\scriptscriptstyle {2}} , x^{\scriptscriptstyle {3}} )\,, \end{aligned}$$

and:

$$\begin{aligned} \phi _{S} ( s^{\scriptscriptstyle {1}} , s^{\scriptscriptstyle {2}} )=\left( \frac{2 s^{\scriptscriptstyle {1}} }{1+( s^{\scriptscriptstyle {1}} )^2+( s^{\scriptscriptstyle {2}} )^2}, \frac{2 s^{\scriptscriptstyle {2}} }{1+( s^{\scriptscriptstyle {1}} )^2+( s^{\scriptscriptstyle {2}} )^2}, \frac{-1+( s^{\scriptscriptstyle {1}} )^2+( s^{\scriptscriptstyle {2}} )^2}{1+( s^{\scriptscriptstyle {1}} )^2+( s^{\scriptscriptstyle {2}} )^2}\right) =( x^{\scriptscriptstyle {1}} , x^{\scriptscriptstyle {2}} , x^{\scriptscriptstyle {3}} )\,. \end{aligned}$$

We proceed by discretizing the unit disk as reference domain once and for all for both hemispheres, with a polygonal approximation of the boundary. We then use the appropriate charts to express the VEM linear and bilinear forms in the northern and southern cells. We connect the two domains together by means of a simple Jacobi domain decomposition approach, and to avoid iterations we use the manufactured solution as boundary condition at the domain interface. Note that the use of curved edges would allow to solve the problem without the need to decompose the computational domain, exploiting the fact that the transition map for the two charts is readily available and sufficiently regular.

In all the experiments, numerical errors are evaluated by defining a manufactured solution $u( s^{\scriptscriptstyle {1}} , s^{\scriptscriptstyle {2}} ):\Omega \rightarrow \mathbb {R}$ and calculating the resulting forcing function $f( s^{\scriptscriptstyle {1}} , s^{\scriptscriptstyle {2}} )$ by substitution into the original equation. Using $u( s^{\scriptscriptstyle {1}} , s^{\scriptscriptstyle {2}} )=\sin (2\pi s^{\scriptscriptstyle {1}} )\sin (2\pi s^{\scriptscriptstyle {2}} )$ and taking into account the contributions of the metric $\mathcal {G}$, the general form of $f( s^{\scriptscriptstyle {1}} , s^{\scriptscriptstyle {2}} )$ can be evaluated as:

$$\begin{aligned} f( s^{\scriptscriptstyle {1}} , s^{\scriptscriptstyle {2}} )&= \sin (2\pi s^{\scriptscriptstyle {2}} )\left( \frac{2\pi w_{(1)}\cos (2\pi s^{\scriptscriptstyle {1}} )}{\sqrt{g_{\scriptscriptstyle {11}}}} +\gamma \sin (2\pi s^{\scriptscriptstyle {1}} )\right)\\&+\frac{\pi \sin (2\pi s^{\scriptscriptstyle {1}} )\cos (2\pi s^{\scriptscriptstyle {2}} )\left( g_{\scriptscriptstyle {11}} \dfrac{ \partial g_{\scriptscriptstyle {22}}}{ \partial s^{\scriptscriptstyle {2}} }-\dfrac{ \partial g_{\scriptscriptstyle {11}}}{ \partial s^{\scriptscriptstyle {2}} }g_{\scriptscriptstyle {22}}\right) }{g_{\scriptscriptstyle {22}}{\text {det}} \mathcal {G} }\\&+\pi \sin (2\pi s^{\scriptscriptstyle {2}} )\left[ \frac{ \cos (2\pi s^{\scriptscriptstyle {1}} )\left( \dfrac{ \partial g_{\scriptscriptstyle {11}}}{ \partial s^{\scriptscriptstyle {1}} } g_{\scriptscriptstyle {22}}-g_{\scriptscriptstyle {11}} \dfrac{ \partial g_{\scriptscriptstyle {22}}}{ \partial s^{\scriptscriptstyle {1}} }\right) }{g_{\scriptscriptstyle {11}}{\text {det}} \mathcal {G} }+ \frac{4\pi \sin (2\pi s^{\scriptscriptstyle {1}} )(g_{\scriptscriptstyle {11}}+g_{\scriptscriptstyle {22}})}{g_{\scriptscriptstyle {22}}{\text {det}} \mathcal {G} }\right]\\&+\frac{2\pi w_{(2)}\sin (2\pi s^{\scriptscriptstyle {1}} )\cos (2\pi s^{\scriptscriptstyle {2}} )}{\sqrt{g_{\scriptscriptstyle {22}}}}, \end{aligned}$$

where $$\hat{\mathbf {w}}=[w_{(1)},w_{(2)}]^{{\tiny T}}$$.

### Test case 1

**Fig. 3:**
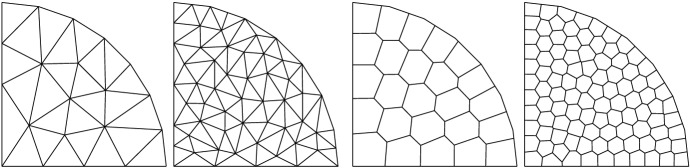
TC1: Level 0 and 1 triangulations (left panels) and polygonal meshes (right panels) of $\Omega$

We use two families of polygonal meshes discretizing the domain (see Fig. 3). To avoid geometric error in the refinement process we uniformly distribute 8 nodes on the curvilinear boundary of $\Omega$ and approximate it with linear interpolation. All the refined meshes are built on this geometry. The meshes in the first family are constrained Delaunay triangulations obtained using Triangle [59, 60], dividing by a factor 4 the area target of the elements at each level. The second family of meshes is obtained by means of PolyMesher [61] by imposing approximately the same number of elements of the triangulation at each corresponding level. Note that the sides of the boundary elements may contain as extra node one of the fixed vertices used to define the curvilinear boundary, and are thus formed by more than one edge.

**Fig. 4:**
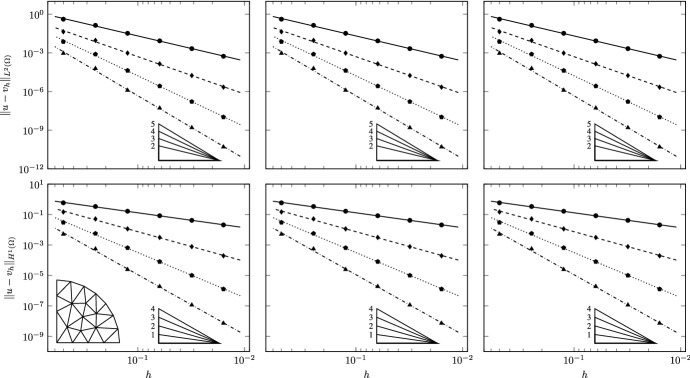
TC1: Convergence of $L^2$ (top) and $H^1$ (bottom) errors vs $h$ on the triangulations. The convergence lines are obtained by approximating via least-squares all the point values. The different lines denote different polynomial orders from 1 (solid line with circular data points) to 4 (dashed-dotted line with triangular data points). The optimal theoretical slope is represented by the lower right triangles

**Fig. 5:**
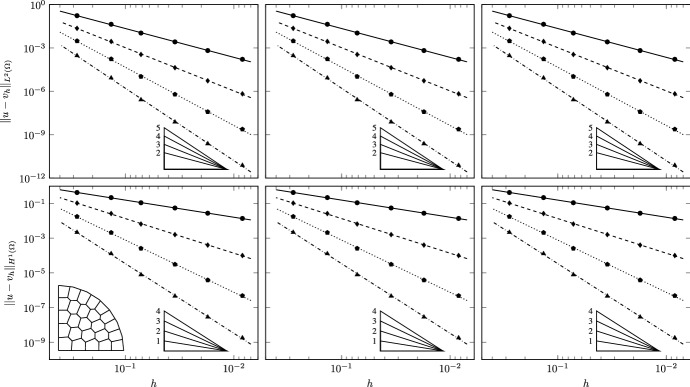
TC1: Convergence of $L^2$ (top) and $H^1$ (bottom) errors vs $h$ on the polygonal meshes. The convergence lines are obtained by approximating via least-squares all the point values. The different lines denote different polynomial orders from 1 (solid line with circular data points) to 4 (dashed-dotted line with triangular data points). The optimal theoretical slope is represented by the lower right triangles

Convergence is tested on four different grid refinement levels, in the cases of $a=0$ and $r=1.1, 1.01, 1.001$. Correspondingly, the results are reported in Figs. 4 and 5 for the triangulations and the polygonal meshes, respectively. The experimental convergence rates are optimal for all the tested polynomial orders, as can be seen from the figures and from the slopes of the lines, which are obtained by approximating via least-squares all the point values. This confirms the theoretical expectations of the behavior of the VEM.

### Test case 2

**Fig. 6:**
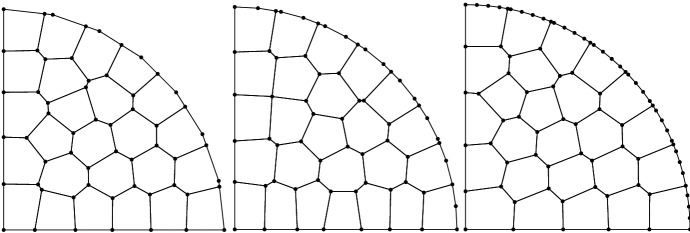
TC2: Level 0, 1, and 2 of polygonal meshes of $\Omega$ with 25 cells and increased boundary resolution (8, 16, and 32 nodes)

This test case is designed to verify the robustness of the scheme for increasingly accurate approximations of the curvilinear boundary. To this aim we look at the errors for a fixed mesh size and vary the number of vertices used to discretize the curvilinear boundary, using only the polygonal mesh. Two sets of meshes are defined: one with approximately 25 elements and the other with 100 elements. In each set we consider 5 mesh levels characterized by different approximations of the curvilinear boundary. In the first level the boundary is discretized with 8 vertices, as in the previous test case. The subsequent levels are obtained by doubling each time the number of nodes located on the curvilinear boundary to arrive at the final level with 128 vertices (see, e.g., Fig. 6). Since the size of the cells remains approximately the same, the boundary sides of the boundary elements are formed by an increasing number of straight edges. While the mesh levels approximate the curvilinear boundary with increasing accuracy, the fact that the length of the edges of the boundary elements becomes unbalanced may lead to increased errors in the VEM solution. However, we know from the literature [23] that such unbalance may only affect the constant that appears in the error estimates. Such constant is increased by a factor proportional to the square root of $\log (1+\ell )$, $\ell$ being the ratio between the maximum and the minimum edge length. Hence, in our experiments we expect the errors to remain approximately constant as we refine the boundary.

**Fig. 7:**
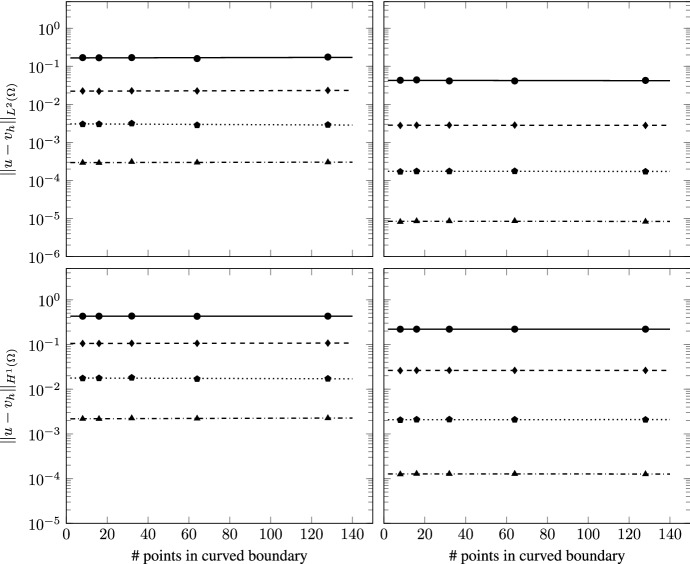
TC2: $L^2$ (top) and $H^1$ (bottom) errors vs number of points on the curvilinear boundary on the polygonal meshes (average of 25 cells, left panels; average of 100 cells, right panels). The different lines denote different polynomial orders from 1 (solid line with circles) to 4 (dashed-dotted line with triangles)

The results of the simulations are shown in Fig. 7, where we report the $L^2$ and $H^1$ errors (top and bottom, respectively) with respect to the manufactured solutions as a function of the number of points discretizing the curved boundary for the two set of meshes (left and right). It is evident that the proposed scheme is robust with respect the increasing unbalance of the edge lengths as the errors for each polynomial order remain approximately constant.

### Test case 3

This test case is aimed at studying the robustness of the scheme to spatial variability and strong anisotropy of the diffusion bilinear form with spatially variable anisotropy ratios. We verify convergence of the proposed VEM scheme by solving our equations on the same families of meshes of Test Case 1 with $r=2$, $k=5$ and two different values for *a*, $a=0.5$ and $a=2$. Figure 2, second and third rows, shows the surface and the spatial distribution of $g_{\scriptscriptstyle {ii}}$ for $k=5$ and $a=0.5$ and 2, respectively. The spatial variability of the anisotropy ratio (ratio between $g_{\scriptscriptstyle {11}}$ and $g_{\scriptscriptstyle {22}}$ or its inverse) varies between 1 and 150 for $a=2$ corresponding to large basis vectors for the tangent plane. This test case challenges the ability of the discretization scheme to handle large and spatially varying anisotropy ratios.

**Fig. 8:**
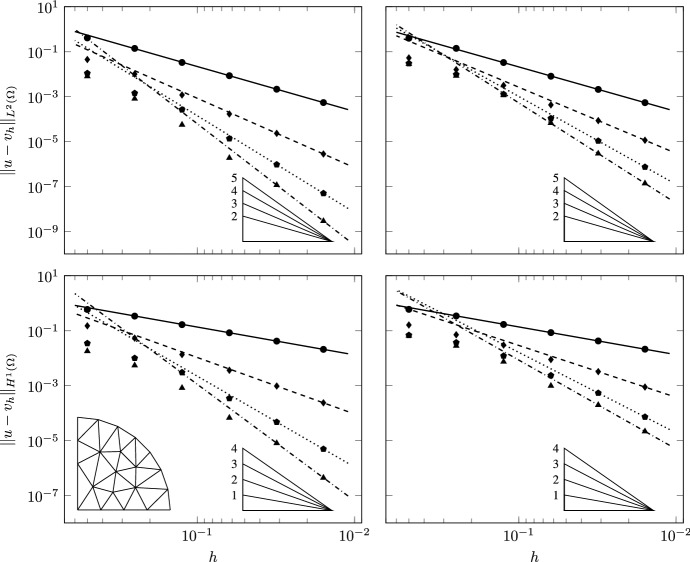
TC3: Convergence of $L^2$ (top) and $H^1$ (bottom) errors versus $h$ on the triangulations ($a=0.5$, left panels; $a=2$, right panels). The convergence lines are obtained by approximating via least-squares only the last two point values. The different lines denote different polynomial orders from 1 (solid line with circles) to 4 (dashed-dotted line with triangles). The optimal theoretical slope is represented by the lower right triangles

**Fig. 9:**
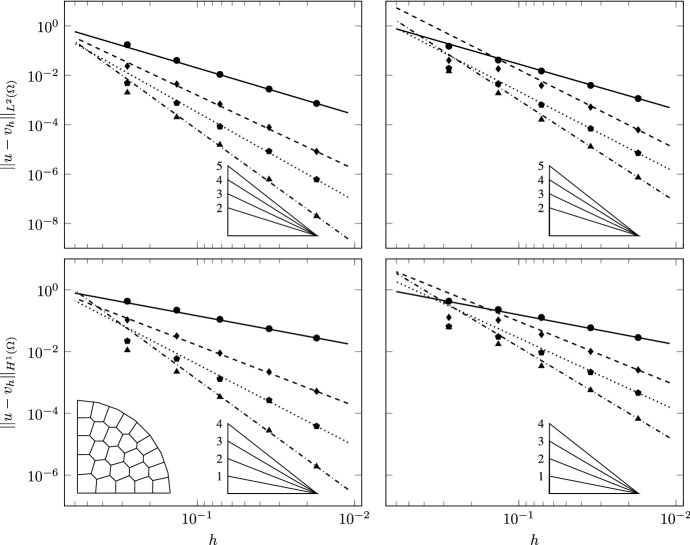
TC3: Convergence of $L^2$ (top) and $H^1$ (bottom) errors vs $h$ on the polygonal meshes ($a=0.5$, left panels; $a=2$, right panels). The convergence lines are obtained by approximating via least-squares only the last two point values. The different lines denote different polynomial orders from 1 (solid line with circles) to 4 (dashed-dotted line with triangles). The optimal theoretical slope is represented by the lower right triangles

Figures 8 and 9 show the numerical convergence of the $L^2$ and $H^1$ norms of the error as a function of $h$. We note that asymptotic behavior of the error is reached as soon as the mesh size is able to resolve the spatial scales of variation of the metric tensor. For this reason the convergence lines in the figures are obtained by interpolation of the last two point values for each polynomial order. Pre-asymptotic convergence is more evident for the higher order polynomials. We attribute this behavior to the smoothing effects of a lower order interpolation. Indeed, the first two or three point values for all polynomial orders display at least the same convergence order of linear polynomials.

**Table 1:** TC3: experimental errors and convergence rates for the triangular mesh set

$\ell$	$\mathcal {P}_{1}$		$\mathcal {P}_{2}$		$\mathcal {P}_{3}$		$\mathcal {P}_{4}$	
	$||u-v_{h}||_{L^2}$	$\hbox {eoc}_{\ell }$	$||u-v_{h}||_{L^2}$	$\hbox {eoc}_{\ell }$	$||u-v_{h}||_{L^2}$	$\hbox {eoc}_{\ell }$	$||u-v_{h}||_{L^2}$	$\hbox {eoc}_{\ell }$
$$a=0.5$$								
0	$4.09\,10^{-1}$	–	$4.47\,10^{-2}$	–	$1.09\,10^{-2}$	–	$8.03\,10^{-3}$	–
1	$1.39\,10^{-1}$	1.556	$1.00\,10^{-2}$	2.154	$1.42\,10^{-3}$	2.943	$8.08\,10^{-4}$	3.313
2	$3.30\,10^{-2}$	2.076	$1.18\,10^{-3}$	3.086	$2.62\,10^{-4}$	2.436	$5.60\,10^{-5}$	3.851
3	$8.50\,10^{-3}$	1.956	$1.69\,10^{-4}$	2.809	$1.35\,10^{-5}$	4.276	$1.86\,10^{-6}$	4.911
4	$2.13\,10^{-3}$	1.995	$2.32\,10^{-5}$	2.861	$9.47\,10^{-7}$	3.837	$1.16\,10^{-7}$	4.000
5	$5.43\,10^{-4}$	1.998	$2.80\,10^{-6}$	3.088	$4.99\,10^{-8}$	4.303	$2.89\,10^{-9}$	5.399
$$a=2.0$$								
0	$3.95\,10^{-1}$	–	$5.26\,10^{-2}$	–	$2.99\,10^{-2}$	–	$2.84\,10^{-2}$	–
1	$1.40\,10^{-1}$	1.495	$1.59\,10^{-2}$	1.730	$9.61\,10^{-3}$	1.639	$8.40\,10^{-3}$	1.755
2	$3.28\,10^{-2}$	2.094	$3.14\,10^{-3}$	2.335	$1.28\,10^{-3}$	2.913	$1.16\,10^{-3}$	2.852
3	$8.07\,10^{-3}$	2.023	$4.27\,10^{-4}$	2.878	$1.07\,10^{-4}$	3.571	$6.66\,10^{-5}$	4.125
4	$2.09\,10^{-3}$	1.951	$8.55\,10^{-5}$	2.321	$1.07\,10^{-5}$	3.324	$2.92\,10^{-6}$	4.512
5	$5.38\,10^{-4}$	1.981	$1.14\,10^{-5}$	2.940	$7.38\,10^{-7}$	3.910	$1.38\,10^{-7}$	4.462

**Table 2:** TC3: experimental errors and convergence rates for the polygonal mesh set

$\ell$	$\mathcal {P}_{1}$		$\mathcal {P}_{2}$		$\mathcal {P}_{3}$		$\mathcal {P}_{4}$	
	$||u-v_{h}||_{L^2}$	$\hbox {eoc}_{\ell }$	$||u-v_{h}||_{L^2}$	$\hbox {eoc}_{\ell }$	$||u-v_{h}||_{L^2}$	$\hbox {eoc}_{\ell }$	$||u-v_{h}||_{L^2}$	$\hbox {eoc}_{\ell }$
$$a=0.5$$								
0	$1.73\,10^{-1}$	–	$2.34\,10^{-2}$	–	$4.84\,10^{-3}$	${-}$	$2.01\,10^{-3}$	–
1	$4.01\,10^{-2}$	2.023	$4.42\,10^{-3}$	2.305	$7.54\,10^{-4}$	2.568	$2.02\,10^{-4}$	3.177
2	$1.08\,10^{-2}$	2.069	$6.83\,10^{-4}$	2.953	$8.36\,10^{-5}$	3.479	$1.54\,10^{-5}$	4.072
3	$2.78\,10^{-3}$	1.888	$7.81\,10^{-5}$	3.012	$8.40\,10^{-6}$	3.191	$6.15\,10^{-7}$	4.471
4	$7.26\,10^{-4}$	1.929	$8.23\,10^{-6}$	3.235	$6.05\,10^{-7}$	3.782	$2.00\,10^{-8}$	4.924
5	$1.81\,10^{-4}$	1.890	$9.07\,10^{-7}$	3.000	$4.33\,10^{-8}$	3.587	$6.78\,10^{-10}$	4.604
$$a=2.0$$								
0	$1.50\,10^{-1}$	–	$4.03\,10^{-2}$	–	$1.92\,10^{-2}$	${-}$	$1.46\,10^{-2}$	–
1	$4.14\,10^{-2}$	1.780	$1.83\,10^{-2}$	1.092	$4.52\,10^{-3}$	2.002	$1.88\,10^{-3}$	2.836
2	$1.49\,10^{-2}$	1.613	$3.87\,10^{-3}$	2.455	$6.31\,10^{-4}$	3.115	$1.61\,10^{-4}$	3.891
3	$3.92\,10^{-3}$	1.859	$5.16\,10^{-4}$	2.799	$6.99\,10^{-5}$	3.056	$1.31\,10^{-5}$	3.486
4	$1.15\,10^{-3}$	1.759	$6.20\,10^{-5}$	3.046	$7.07\,10^{-6}$	3.294	$7.32\,10^{-7}$	4.142
5	$2.99\,10^{-4}$	1.832	$5.83\,10^{-6}$	3.214	$6.30\,10^{-7}$	3.290	$3.52\,10^{-8}$	4.130

The high degree of anisotropy of this test case causes a loss of convergence in the higher polynomial orders. This behavior is more evident for the polygonal meshes. To better quantify this convergence loss, Tables 1 and 2 report the convergence order sequence for the triangulations and polygonal meshes, respectively. We note that, for first and second order polynomials, almost optimal order of convergence is reached for both mesh sets and both $a=0.5$ and $a=2$. In the higher orders, a clear loss of at least half an order is evident. This can be attributed to difficulties in resolving the large anisotropy ratios that are typical of this test case. The fact that this occurs only for the higher orders is due to the ill-conditioning of the resulting linear system that controls the ratio between the norms of the residuals and the errors in the linear system solver.

### Test case 4

**Fig. 10:**
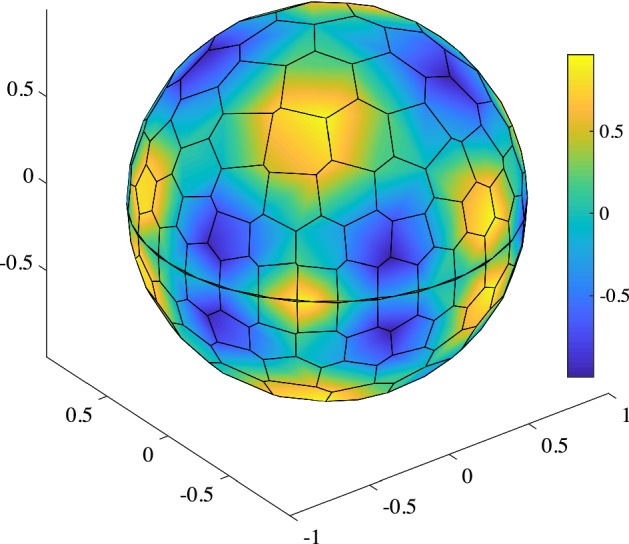
TC4: Numerical solution (nodal dofs only) on the coarsest mesh with linear interpolation from the nodal values

This test case shows the convergence properties of the proposed framework in a multiple charts setting. We discretize the full unit disk with 5 polygonal mesh levels following the strategy reported in Test Case 1, so that the level $\ell =0$ is characterized by 100 cells and the last one ($\ell =4$) by 25600 cells. The VEM solution, reconstructed on $\varGamma =S^2$ using nodal only degrees of freedom, is shown in Fig. 10 for the coarsest mesh.

**Fig. 11:**
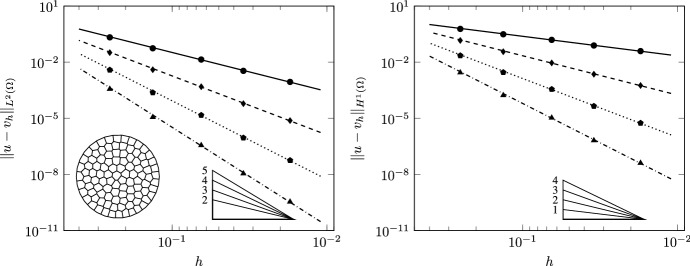
TC4: Convergence of $L^2$ (left) and $H^1$ (right) errors versus $h$ on the polygonal meshes. The convergence lines are obtained by approximating via least-squares only the last three point values. The different lines denote different polynomial orders from 1 (solid line with circles) to 4 (dashed-dotted line with triangles). The optimal theoretical slope is represented by the lower right triangles

**Table 3:** TC4: experimental errors and convergence rates for the polygonal mesh set

$\ell$	$\mathcal {P}_{1}$		$\mathcal {P}_{2}$		$\mathcal {P}_{3}$		$\mathcal {P}_{4}$	
	$||u-v_{h}||_{L^2}$	$\hbox {eoc}_{\ell }$	$||u-v_{h}||_{L^2}$	$\hbox {eoc}_{\ell }$	$||u-v_{h}||_{L^2}$	$\hbox {eoc}_{\ell }$	$||u-v_{h}||_{L^2}$	$\hbox {eoc}_{\ell }$
0	$2.12 \,10^{-1}$	–	$3.23 \,10^{-2}$	–	$3.85 \,10^{-3}$	–	$3.68 \,10^{-4}$	–
1	$5.52 \,10^{-2}$	2.104	$3.96 \,10^{-3}$	3.284	$2.36 \,10^{-4}$	4.367	$1.1510^{-5}$	5.424
2	$$ 1.38 \,10^{-2}$$	1.924	$$ 4.88 \,10^{-4}$$	2.905	$$ 1.4710^{-5}$$	3.857	$$ 3.61 \,10^{-7} $$	4.801
3	$$ 3.42 \,10^{-3}$$	2.221	$$ 6.0510^{-5}$$	3.326	$$ 9.02 \,10^{-7}$$	4.443	$$ 1.11 \,10^{-8} $$	5.543
4	$8.78 \,10^{-4}$	1.960	$7.53 \,10^{-6}$	3.002	$5.63 \,10^{-8}$	3.995	$3.44 \,10^{-10}$	5.009

The experimental convergence on these mesh levels is reported for the $L^2$ and $H^1$ norms of the error as a function of $h$ in Fig. 11 and in Table 3. The numerical results show that the proposed approach is functioning as expected and that the use of two different charts doe not influence the optimal convergence of the scheme. Obviously, this is a very favorable case as the stereographical projection produces charts and, if necessary, transition maps that are sufficiently smooth to allow high order. In the future it will be important to study how to derive charts and transition maps with specified regularity for different surfaces, possibly starting from the work of [44].

## Conclusions

We have developed an arbitrary-order virtual element method for the discretization of elliptic surface PDEs. The approach employs a local parametrization of the surface to properly re-define the PDE on the local chart. This allows the straight-forward definition of a two-dimensional VEM discretization at all polynomial orders, overcoming the difficult task of the consistent approximation of the surface and of the distance function of its tubular neighborhood. The drawback of the approach is that the geometrically intrinsic form of the PDE contains the metric information, which may be strongly non-isotropic and highly variable in space, depending the regularity of the surface. The choice of the VEM scheme is motivated by the need to ensure robustness and high order of convergence for these anisotropic and spatially variable coefficients.

The developed scheme has been tested on several numerical examples showing varying degrees of regularity. In fact, optimal orders of convergence up to 5 has been reached for surfaces with relatively small curvatures. Only when curvatures and metric information become extremely large loss of convergence is noticed. This loss of convergence is related to the presence of strongly anisotropic diffusion tensors and strongly aligned advective fields due to the behavior of the metric tensor. Handling strong anisotropy is still a major challenge in the numerical solution of PDEs by the virtual element method, and is left for future research. This difficulty can be relaxed also by employing multiple charts that decrease the anisotropic characteristic of the metric tensor. However, proper regularity of the transition maps between the different charts must be ensured to achieve full order convergence. To verify the ability of our formulation to work with multiple charts we tested the proposed scheme on the full sphere by employing two charts arising from the stereographical projection. Future work will be addressed to define for general surfaces appropriate multiple charts with regular transition maps starting from the work of [44].

One of the major advantages of the developed VEM formulation is that can be used efficiently to minimize geometric errors of curvilinear boundaries. We have tested our approach on an hemispherical surface discretized by a fixed number of polygonal cells, where the boundary edges formed by an increasing number of nodes. The resulting errors were independent of the edge discretization, showing the robustness of the VEM scheme in this situation.
